# Supplementary material for: Hemiparasitic plants increase alpine plant richness and evenness but reduce arbuscular mycorrhizal fungal colonization in dominant plant species
Source: PeerJ. 2018 Nov 7;6:e5682. doi: 10.7717/peerj.5682 (PMC6228546; doi:10.7717/peerj.5682)
Supplement: Supplemental Information 1 — Full mixed models were constructed with Castilleja presence and elevation as fixed factors and intercepts were allowed to vary by plot pairings (random effect). Fixed effects were sequentially removed from the full model until we only fit the random effect. We report the number of model parameters (df) and AIC score (AIC). [file peerj-06-5682-s001.docx]

Table S1: Mixed-effect model selection for plant richness, plant evenness (Probability of Interspecific Encounter), mycorrhizal fungal colonization, and dark-septate endophyte colonization data. Plot pairings were included as a random effect for all models.

Plant Richness df AIC

**Richness ~ *Castilleja* presence × Elevation 6 406.816**

Richness ~ *Castilleja* presence 4 432.960

Richness ~ Elevation 4 406.816

Richness ~ 1 3 406.816

Plant Evenness df AIC

**Evenness ~ *Castilleja* presence × Elevation 6 -155.607**

Evenness ~ *Castilleja* presence 4 -129.712

Evenness ~ Elevation 4 -151.6243

Evenness ~ 1 3 -125.272

Mycorrhizal colonization df AIC

Myc ~ *Castilleja* presence × Elevation 6 656.835

**Myc ~ *Castilleja* presence 4 653.620**

Myc ~ Elevation 4 750.721

Myc ~ 1 3 748.946

Dark-septate endophyte colonization df AIC

DSE ~ *Castilleja* presence × Elevation 6 796.831

DSE ~ *Castilleja* presence 4 804.511

**DSE ~ Elevation 4 795.823**

DSE ~ 1 3 804.875
