# Supplementary material for: Hemiparasitic plants increase alpine plant richness and evenness but reduce arbuscular mycorrhizal fungal colonization in dominant plant species
Source: PeerJ. 2018 Nov 7;6:e5682. doi: 10.7717/peerj.5682 (PMC6228546; doi:10.7717/peerj.5682)
Supplement: Supplemental Information 2 — Df = degrees of freedom, SS = Sequential sums of Squares, MeanSqs = Mean squares, F = F statistic, R2 = partial R2, P = p value. [file peerj-06-5682-s002.docx]

Table S2:

| Factor | Df | SS | | MeanSqs | | *F* | | R^2^ | | *P* | |  |
| --- | --- | --- | --- | --- | --- | --- | --- | --- | --- | --- | --- | --- |
| Elevation | 1 | | 7.852 | | 7.8519 | | 33.936 | | 0.2100 | | 0.01 | |
| *Castilleja* present | 1 | | 0.791 | | 0.7907 | | 3.418 | | 0.0211 | | 0.01 | |
| Plot pairing | 1 | | 3.374 | | 3.3739 | | 14.582 | | 0.0902 | | 0.01 | |
| Elevation × *Castilleja* present | 1 | | 0.586 | | 0.5859 | | 2.532 | | 0.0157 | | 0.01 | |
| Elevation × Pairing | 1 | | 3.119 | | 3.1192 | | 13.481 | | 0.0834 | | 0.01 | |
| *Castilleja* present × Pairing | 1 | | 0.245 | | 0.2450 | | 1.059 | | 0.0065 | | 0.32 | |
| Elevation × *Cas*. pres.× Pairing | 1 | | 0.131 | | 0.1311 | | 0.566 | | 0.0035 | | 0.89 | |
| Residuals | 92 | | 21.287 | | 0.2314 | |  | | 0.5694 | |  | |
| Total | 99 | | 37.384 | |  | |  | | 1.0000 | |  | |
