# Supplementary material for: Hemiparasitic plants increase alpine plant richness and evenness but reduce arbuscular mycorrhizal fungal colonization in dominant plant species
Source: PeerJ. 2018 Nov 7;6:e5682. doi: 10.7717/peerj.5682 (PMC6228546; doi:10.7717/peerj.5682)
Supplement: Supplemental Information 3 — Df = degrees of freedom, SS = Sequential sums of Squares, MeanSqs = Mean squares, F = F statistic, R2 = partial R2, P = p value. [file peerj-06-5682-s003.docx]

Table S3:

| Elevation | Factor | | Df | | SS | | MeanSqs | | *F* | | R^2^ | *P* |
| --- | --- | --- | --- | --- | --- | --- | --- | --- | --- | --- | --- | --- |
| 2480m | *Castilleja* present | 1 | | 0.044 | | 0.04392 | | 0.427 | | 0.0220 | | 0.85 |
|  | **Plot pairing** | **1** | | **0.279** | | **0.27928** | | **2.714** | | **0.1399** | | **0.02** |
|  | *Cas.* present × Pairing | 1 | | 0.026 | | 0.02596 | | 0.252 | | 0.0130 | | 0.90 |
|  | Residuals | 16 | | 1.646 | | 0.10290 | |  | | 0.8250 | |  |
|  | Total | *19* | | 1.996 | |  | |  | | 1.0000 | |  |
|  |  |  | |  | |  | |  | |  | |  |
| 2740m | *Castilleja* present | 1 | | 0.125 | | 0.12511 | | 1.106 | | 0.0571 | | 0.38 |
|  | Plot pairing | 1 | | 0.185 | | 0.18517 | | 1.637 | | 0.0846 | | 0.13 |
|  | *Cas.* present × Pairing | 1 | | 0.070 | | 0.06964 | | 0.616 | | 0.0318 | | 0.77 |
|  | Residuals | 16 | | 1.810 | | 0.11311 | |  | | 0.8265 | |  |
|  | Total | *19* | | 2.190 | |  | |  | | 1.0000 | |  |
|  |  |  | |  | |  | |  | |  | |  |
| 3200m | *Castilleja* present | 1 | | 0.141 | | 0.14051 | | 1.653 | | 0.0832 | | 0.20 |
|  | Plot pairing | **1** | | 0.133 | | 0.13341 | | 1.570 | | 0.0790 | | 0.20 |
|  | *Cas.* present × Pairing | 1 | | 0.054 | | 0.05451 | | 0.641 | | 0.0323 | | 0.69 |
|  | Residuals | 16 | | 1.360 | | 0.08500 | |  | | 0.8054 | |  |
|  | Total | *19* | | 1.688 | |  | |  | | 1.0000 | |  |
|  |  |  | |  | |  | |  | |  | |  |
| 3392m | *Castilleja* present | 1 | | 0.061 | | 0.06075 | | 1.000 | | 0.0491 | | 0.45 |
|  | Plot pairing | 1 | | 0.120 | | 0.11972 | | 1.971 | | 0.0968 | | 0.08 |
|  | *Cas.* present × Pairing | 1 | | 0.085 | | 0.08506 | | 1.401 | | 0.0688 | | 0.18 |
|  | Residuals | 16 | | 0.972 | | 0.06072 | |  | | 0.7854 | |  |
|  | Total | *19* | | 1.237 | |  | |  | | 1.0000 | |  |
|  |  |  | |  | |  | |  | |  | |  |
| 3460m | *Castilleja* present | 1 | | 0.090 | | 0.09031 | | 0.695 | | 0.0360 | | 0.64 |
|  | Plot pairing | 1 | | 0.286 | | 0.28609 | | 2.202 | | 0.1142 | | 0.06 |
|  | *Cas.* present × Pairing | 1 | | 0.051 | | 0.05064 | | 0.390 | | 0.0202 | | 0.84 |
|  | Residuals | 16 | | 2.079 | | 0.12992 | |  | | 0.8296 | |  |
|  | Total | *19* | | 2.506 | |  | |  | | 1.0000 | |  |
